# Case report: Pulmonary non-Langerhans cell histiocytosis in a dog with acute interstitial granulomatous pneumonia

**DOI:** 10.3389/fvets.2025.1522119

**Published:** 2025-02-25

**Authors:** Kyle L. Granger, Kurt Williams, Alex Ohlendorf, Sarah Shropshire, Kelly E. Hall

**Affiliations:** ^1^Department of Clinical Sciences, College of Veterinary Medicine and Biomedical Sciences, Colorado State University, Fort Collins, CO, United States; ^2^Department of Biomedical Sciences, Carlson College of Veterinary Medicine, Oregon State University, Corvallis, OR, United States; ^3^Department of Environmental and Radiological Health Sciences, College of Veterinary Medicine and Biomedical Sciences, Colorado State University, Fort Collins, CO, United States

**Keywords:** non-Langerhans cell histiocytosis, acute interstitial granulomatous pneumonia, acute interstitial pneumonia (AIP), interstitial lung disease (ILD), respiratory distress

## Abstract

**Background:**

Pulmonary involvement of Non-Langerhans Cell Histiocytosis (PNLCH) is a rare cause of interstitial pulmonary disease in people and are classified as either Erdheim-Chester disease (ECD) or Rosai-Dorfman disease (RDD). In veterinary medicine, feline pulmonary Langerhans cell histiocytosis (PLCH) has been identified as an infiltrative histiocytic disorder with an insidious onset of progressive respiratory distress and is non-responsiveness to empiric therapies. Unfortunately, subsequent death either from respiratory failure or humane euthanasia are the reported outcomes in all reported cases. To date, a similar primarily pulmonary histiocytic disease has not been described in dogs. We present a case of an 8-year-old male intact Rottweiler with acute, progressive respiratory failure with a post-mortem diagnosis of PNLCH.

**Case summary:**

An 8-year-old male intact Rottweiler presented following approximately 2 weeks of lethargy, anorexia, hypersalivation, and progressive respiratory distress characterized by intermittent wheezing, increasing inspiratory and expiratory effort, and tachypnea. Diagnostic imaging demonstrated a multifocal cranioventral alveolar pattern with nodules in the lung periphery. There were no significant changes appreciated in bloodwork. Despite empiric antimicrobials, oxygen support, and other supportive care measures, the patient continued to deteriorate and was subsequently euthanized. Post-mortem analysis was confirmatory for single-organ PNLCH.

**New or unique information provided:**

This case report represents the first reported case of canine PNLCH. Additionally, this report also provides further characterization of PNLCH in dogs with ante-mortem diagnostic imaging, cytologic evaluation of lung tissue, and post-mortem immunohistochemical characterization of canine PNLCH.

## Introduction

Histiocytic proliferative disorders (HPDs) are well documented in veterinary medicine. HPDs are derived from an overproliferation of histiocytes, an overarching term to describe cells of either macrophage or dendritic cell lineage (1–4). In dogs, HPDs are currently classified into three major groups: reactive histiocytosis, cutaneous histiocytoma complex, and histiocytic sarcoma complex (1, 2). Their classification is largely dependent on the clinical behavior of the disease and their histopathologic features. While histiocytic diseases involving Langerhans cells (LC) are well documented as histiocytomas and cutaneous LC histiocytosis (LCH) in dogs, non-Langerhans cell histiocytosis (NLCH) are not yet reported.

Unlike pyogranulomatous bronchopneumonia, which often presents with infectious agents and neutrophilic inflammation predominating (5), NLCH is characterized by distinct histiocytic infiltrates that lack an infectious etiology and primarily involve macrophages and dendritic cells. This differentiation is essential for understanding this novel presentation in veterinary species.

In people, pulmonary manifestations of NLCH are rare and more commonly diagnosed as either Erdheim-Chester disease (ECD) or Rosai-Dorfman disease (RDD) in adults and juvenile xanthogranuloma (JXG) in children (6). Currently, in people, HPDs are only distinguishable through immunohistochemistry (IHC) performed on lung biopsy tissue or extranodal sites (7, 8). To the best of our knowledge, this is the first reported case of pulmonary NLCH in a dog.

## Case presentation

An 8-year-old male intact Rottweiler presented to the Urgent Care service of Colorado State University (CSU) in September 2022 as a referral case from a nearby emergency facility. The owners reported in early September 2022, the dog was lethargic, hypo-to anorexic, and would intermittently wheeze and the dog’s respiratory rate would progressively increase and hypersalivation was noted. The dog was presented to the referring veterinarian where thoracic radiographs revealed an interstitial to alveolar pattern, suspected to be due to aspiration pneumonia from the previously noted hypersalivation. It should be noted that approximately both 2 and 4 months prior, the dog had normal thoracic radiographs with the primary care veterinarian. A point-of-care chemistry panel, including electrolytes, glucose, and renal markers, was performed and found to be reportedly unremarkable. An abdominal ultrasound documented unchanged segmental thickening of the small intestine and unchanged splenic nodules that had been noted 4 months prior with the primary care veterinarian. The dog was placed in an oxygen chamber with intravenous fluid therapy, ampicillin/sulbactam, enrofloxacin, and maropitant of unknown dosages for 2 days. The dog was not perceived to be improving and was transferred to the CSU Veterinary Teaching Hospital (VTH) for further investigation and treatment.

**Figure 1:**
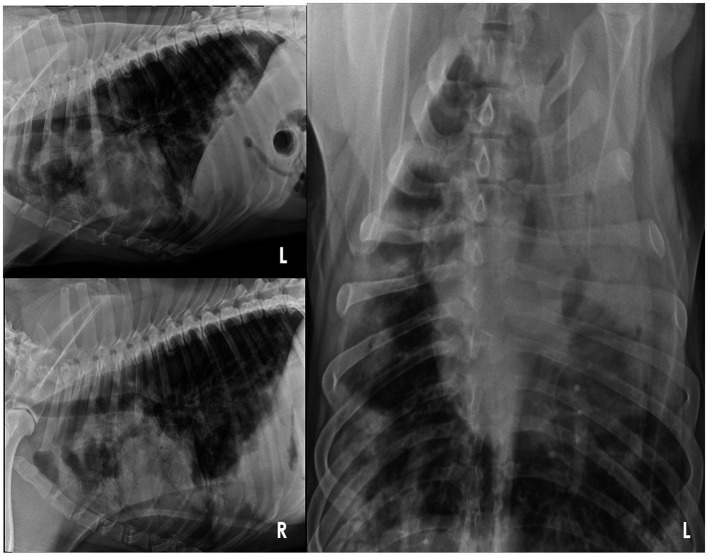
Three-view thoracic radiographs of an 8-year-old Rottweiler with pulmonary non-Langerhans cell histiocytosis. Note the numerous, poorly marginated, variably sized patchy regions of alveolar patterns throughout the pulmonary periphery, some of which have a nodular appearance. L, left; R, right.

**Figure 2:**
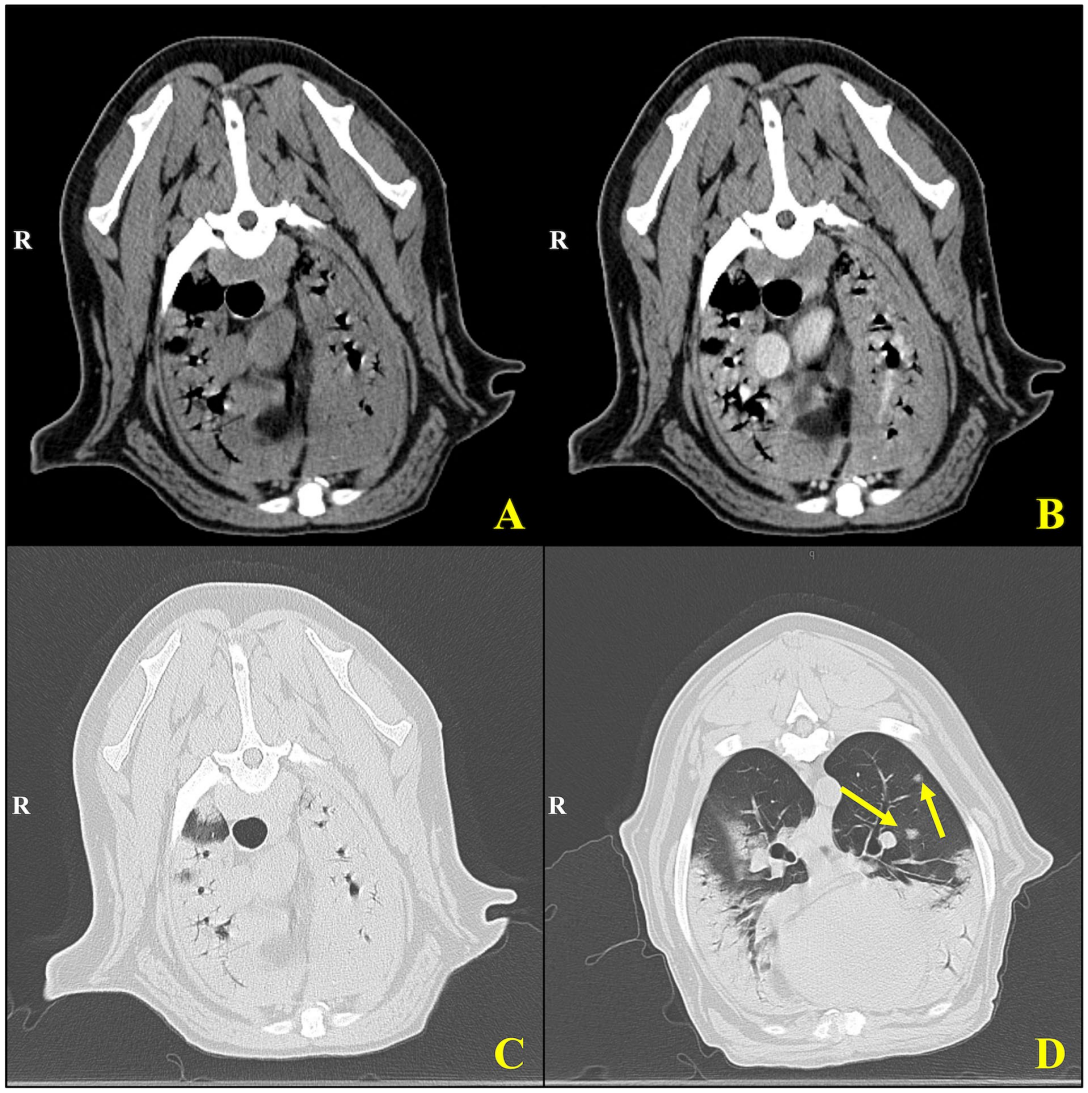
Cross-sectional computed tomography of an 8-year-old Rottweiler with pulmonary non-Langerhans cell histiocytosis. Transverse CT images of the thorax using soft tissue **(A,B)** and lung window settings **(C,D)**. **(A)** Pre-contrast and **(B)** post-contrast images demonstrate mediastinal and peribronchial structures with visible vascular landmarks. The post-contrast image **(B)** shows enhanced delineation of mediastinal vasculature and soft tissues, aiding in the assessment of potential lymphadenopathy or vascular abnormalities. **(C,D)** Lung window images provide a detailed view of parenchymal structures, with **(D)** highlighting multiple well-defined pulmonary nodules (yellow arrows) within the cranial and middle lung lobes. These nodules exhibit a random distribution and vary slightly in size, consistent with granulomatous inflammation or metastatic disease processes.

At the VTH, the dog was severely tachypneic with mild hypersalivation, had diffuse harsh lung sounds most prominently noted in the cranioventral pulmonary fields, and had a mild prostatomegaly on rectal examination. An arterial blood gas (ABG) was initially performed, with flow by oxygen, which demonstrated a low pH (7.266, reference interval [RI]: 7.35–7.45), hypercapnia (PCO_2_: 57.3 mmHg, RI: 24 – 39 mmHg), normal absolute base excess (ABE: −2.2, RI: −4 – 4), normal bicarbonate (HCO_3_^−^: 25.2 mEq/L, RI: 17 – 27 mEq/L), normal lactate (1.9, RI: 0-2 mmol/L), hypoxemia (PO_2_: 58.7 mmHg, RI: 70 – 92 mmHg; sO2: 79.7%, RI: 95–100%), which was consistent with an acute primary respiratory acidosis. Additional bloodwork performed consisted of a complete blood count and serum biochemistry panel which revealed a mild leukocytosis (20.1 × 10^3^/mL; RI: 4.5–15 × 10^3^/mL), mild anemia (hematocrit [HCT]: 37%, RI: 40–55%; HGB: 12.5 g/dL, RI: 13 – 20 g/dL), mild hypomagnesemia (Mg^2+^: 1.5 mg/dL, RI: 1.8–2.4 mg/dL), moderate hypoalbuminemia (albumin: 2.2 g/dL, RI: 3.0–4.3 g/dL), normal serum bicarbonate (HCO_3_^−^: 25.3 mEq/L, RI: 15 – 27 mEq/L), and mild hypochloremia (Cl^−^: 105.3 mEq/L, RI: 108-118 mEq/L), and the remaining values were within normal limits. Three-view thoracic radiographs revealed numerous, poorly marginated, variably sized patchy regions throughout the pulmonary periphery, some of which have a nodular appearance (Figure 1). A thoracic and abdominal triple phase CT scan with contrast was performed under general anesthesia which revealed a marked, multifocal patchy regions of soft tissue opacity with air bronchograms with a predominantly ventral distribution throughout the lungs and many, poorly marginated, variably sized patchy to nodular regions throughout the pulmonary periphery (Figure 2). There was also mild sternal, cranial mediastinal, tracheobronchial, and jejunal lymph node enlargement (Supplementary Figure 1). Cystic nodules were also found in the cranioventral liver and head of the spleen, and the prostate was moderately enlarged. A coagulation panel was performed which showcased a mild elevation in activated partial thromboplastin time (aPTT: 14.9 s, RI: 9.8–13.3 s), mildly decreased anti-thrombin concentrations (AT: 73%, RI: 104–162%), and mildly elevated D-Dimer concentrations (0.72 μg/mL, RI: 0.03–0.4 μg/mL) and fibrinogen (612 mg/dL, RI: 123–210 mg/dL) suggestive of non-specific systemic inflammation. Fine-needle aspirates of the lung were obtained which demonstrated moderate mixed inflammation (neutrophilic and histiocytic) with no extra-or intra-cellular bacteria noted, epithelial dysplasia, and atypical mesenchymal proliferation. While recovering from general anesthesia, the dog was sterilely transitioned to mechanical ventilation for continued respiratory support. A recheck ABG was performed while the dog remained intubated and was receiving 100% oxygen supplementation which demonstrated normal pH (7.385, RI: 7.35–7.45), mildly improved hypercapnia (PCO_2_: 42.3 mmHg, RI: 24–39 mmHg), normal absolute base excess (ABE: 0.2, RI: −4 – 4), normal bicarbonate (HCO_3_^−^: 24.8 mEq/L, RI: 17 – 27 mEq/L), normal lactate (1.5, RI: 0 – 2 mmol/L), and mildly improved hypoxemia (PO_2_: 65.2 mmHg, RI: 70 – 92 mmHg; sO_2_: 92.5%, RI: 95–100%). Given the uncertain prognosis and the possibility of an aggressive neoplasia process, the owners elected for humane euthanasia and necropsy.

## Post-mortem analysis

**Figure 3:**
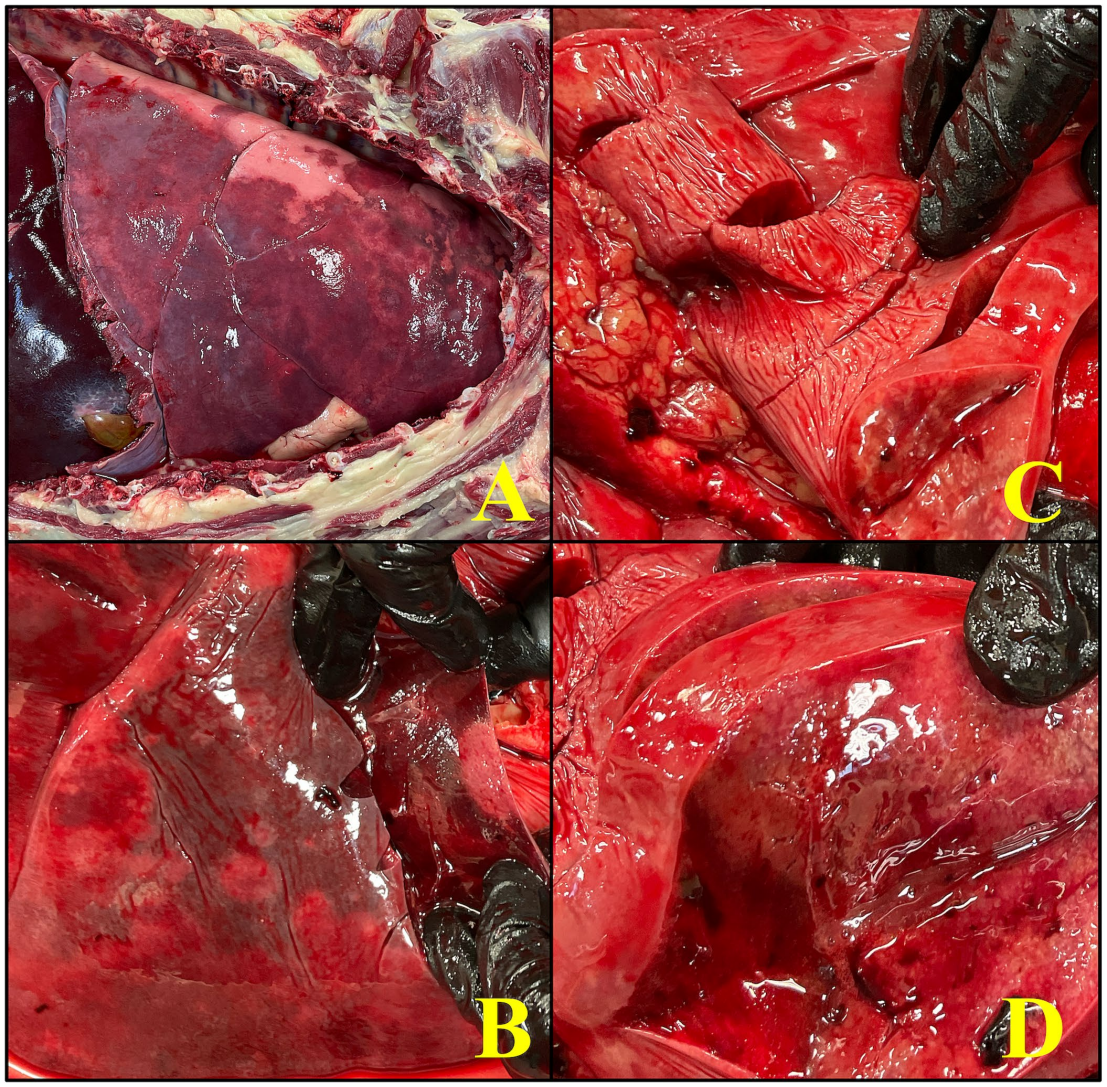
An image series showcases the gross necropsy findings in an 8-year-old male intact Rottweiler diagnosed with pulmonary non-Langerhans cell histiocytosis (PNLCH). **(A)** Picture of the lungs within the thoracic cavity which appreciably has severe discoloration, palpates firm, and has a multifocal tan nodular appearance, consistent with the widespread granulomatous disease. **(B,C)** The lower images demonstrate various stages of dissection, with emphasis on the infiltrative and fibrotic nature of the lesions within the lungs. **(D)** A close up of the lungs on cut section to highlight the presence of coalescing lesions with fibrinous exudate covering the pleura which occupy a significant portion of the pulmonary tissue with a nodule on cut section, characteristic of acute inflammatory response.

On gross evaluation, approximately 95% of the pulmonary parenchyma contained pale-tan nodules measuring 0.5–3 cm (Figure 3). The lungs did not float when placed in 10% formalin. The spleen had greater than 10 dark red to pale tan, firm nodules measuring 0.5–3 cm in diameter. The liver had multifocal regions of suspected capsular fibrosis which did not extend into the underlying parenchyma. The prostate was asymmetrically enlarged with expansion of the caudal aspect of the right lobe by a dark brown, 1 cm nodule.

**Figure 4:**
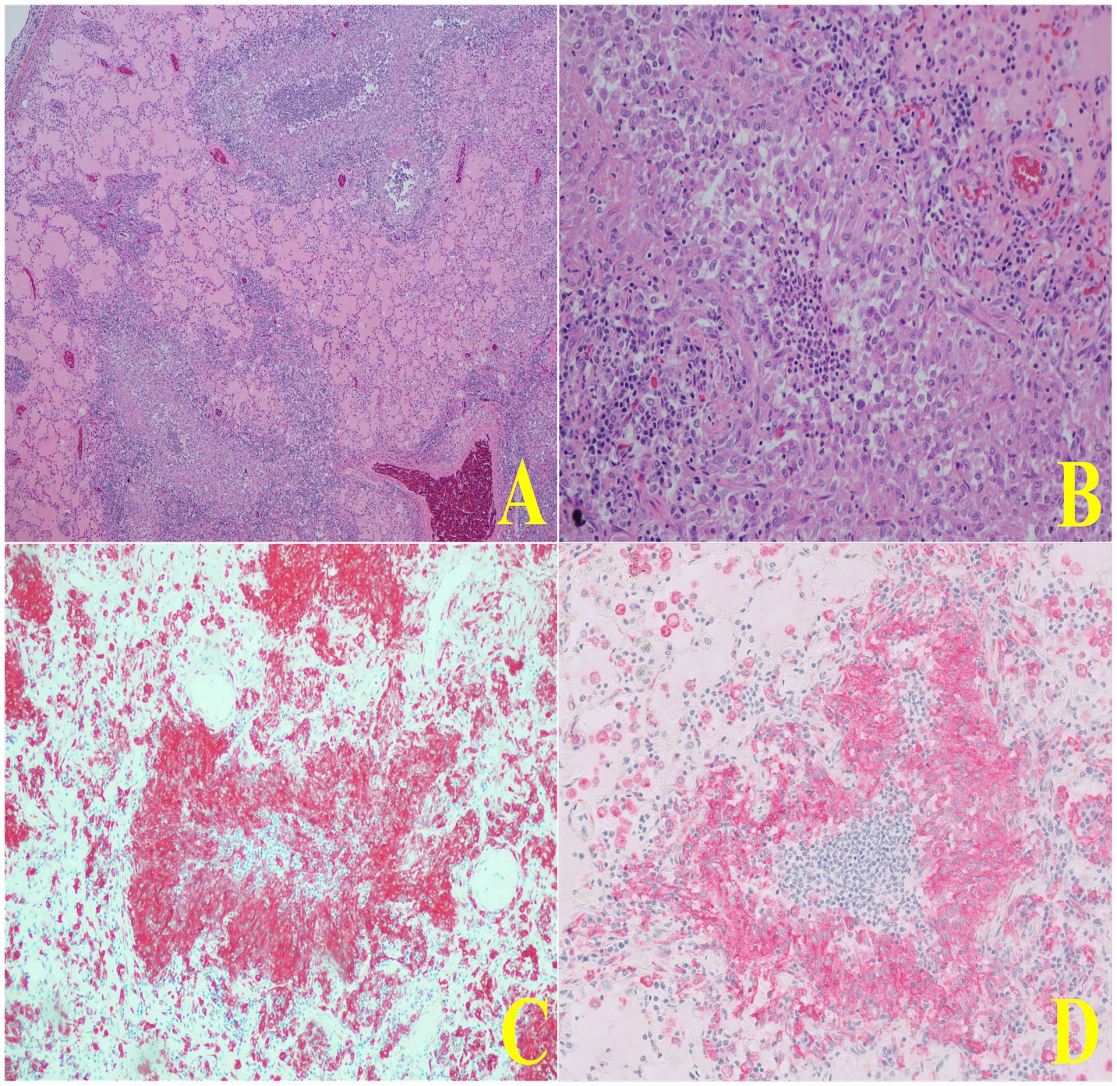
Representative histopathologic images of lung tissue demonstrating macrophage infiltration. In panel **(A)**, a low-magnification H&E-stained section shows two airways with notable macrophage infiltration concentrated within the peribronchial regions. Panel **(B)** presents a higher-magnification view of a single airway, revealing the dense accumulation of macrophages surrounding the bronchial lumen. In panels **(C,D)**, immunohistochemistry staining for CD204 further highlights these macrophages, showing strong positive staining around the same airway, confirming the presence of a significant macrophage population. In panel **(D)**, the granuloma exhibits a central area of degenerate neutrophils and karyorrhectic debris and is lined by epithelioid macrophages and circumferentially surrounded by fibrosis creating mass-effects in the tissue.

Histologically, lung alveoli were diffusely filled with eosinophilic fluid, wispy eosinophilic material (fibrin), marked numbers of non-degenerate neutrophils and centralized foamy and pigment-laden macrophages. The alveolar septa were often effaced by sheets of macrophages and non-degenerate neutrophils (Figure 4). Multifocally the parenchyma was effaced by granulomas with central areas of non-degenerate neutrophils and karyorrhectic debris, lined by epithelioid macrophages and circumferentially surrounded by fibrosis creating mass-like effects in the tissue (Figure 4). Large airways were transmurally infiltrated by lymphocytes, plasma cells, and neutrophils, and the lumen was filled with sloughed epithelium and non-degenerate neutrophils. The pleural surface was markedly and diffusely expanded by many lymphocytes, plasma cells, macrophages and fibrin. The spleen demonstrated benign lymphoid hyperplasia and hemorrhage, the liver exhibited multifocal chronic capsular fibrosis, and the small intestine showcased moderate chronic lymphoplasmacytic enteritis.

Special stains performed on the pulmonary tissue included Giemsa, Grocott-Gömöri methenamine silver stain (GMS), Periodic acid–Schiff (PAS), Ziehl-Neelsen Acid Fast, Luna, Gram, Silver, Site-Acid Fast, and Warthin-Starry; all yielded negative results. Immunohistochemistry was conducted using markers such as e-cadherin, CD3, PAX-5, S-100 protein, and canine distemper virus, which were also negative. Additional IHC stains, including CD204 and IBA-1, demonstrated marked intracytoplasmic reactivity in epithelioid macrophages and alveolar histiocytes. Alveolar histiocytes exhibited marked intracytoplasmic reactivity to factor XIIIa, CD90, and CD163, while epithelioid macrophages were negative for these markers. All stains and methods followed routine protocols. A comprehensive list of antibodies tested, and their results, is provided in Supplementary Table 1.

Additional testing included anaerobic and aerobic cultures of liver tissue; both negative. Lung tissue samples were submitted for canine distemper virus real-time PCR, S*treptococcus zooepidemicus* real-time PCR, influenza A virus real-time PCR, *Bartonella* spp. PCR, *Mycoplasma* spp. with DNA sequencing PCR, *Mycobacterium* spp. real-time PCR, and *Mycobacterium* spp. culture; all of which were negative.

## Discussion

Pulmonary NLCH (PNLCH) is a relatively complex condition in people, characterized by the presence of atypical histiocytic infiltrates within the lungs. In people, NLCH disorders share some common pathways, however, the precise causes are multifactorial, involving a mix of genetic predispositions, immune responses, and potential environmental or infectious triggers (6, 7). In veterinary medicine, NLCH with pulmonary involvement has not been reported. Furthermore, the discussion delves into the potential relationship between PNLCH and acute interstitial granulomatous pneumonia (AIP), a disease not previously explored in veterinary medicine. This case highlights the need for further research to enhance our understanding of PNLCH and develop effective diagnostic and treatment strategies for afflicted animals.

Unlike ECD in people, which can be characterized by systemic involvement of multiple organs including the CNS, cardiovascular system, kidneys, and long bones (7, 9–11), this case was limited to the lungs with no overt evidence of extra-pulmonary involvement. Furthermore, the rapid clinical progression over less than 3 weeks stands in stark contrast to the chronic, indolent course typically observed in people with ECD. Pulmonary involvement in ECD is usually a slowly progressive interstitial disease associated with extensive fibrosis (7, 9–11), whereas this case displayed acute, severe inflammatory changes with minimal fibrosis. However, it is important to note that ECD and other NLCHs can also present with isolated pulmonary involvement (9, 12), particularly in atypical cases reported in human medicine. These differences highlight the potential uniqueness of this case and suggest an alternate pathophysiological process distinct from known NLCH presentations in people. The comparison to these, among other NLCH diseases in people, provides a framework for understanding the immunophenotype of the infiltrate but underscores the need for further study to define this entity in veterinary medicine.

While the pathophysiology of PNLCH in people remains challenging, its clinical manifestations show intriguing parallels to feline PLCH, including rapid progression, bronchiolocentric immune-cell obliteration of the airways, and diffuse pulmonary involvement. However, this dog’s IHC profile does not support a diagnosis of PLCH (Supplementary Table 1). In people, the diagnosis of NLCH is often differentiated from LCH using IHC markers such as CD90 (Thy-1), e-cadherin, and factor XIIIa (FXIIIa). CD90 and FXIIIa are typically expressed in NLCH cases, while e-cadherin is a hallmark of LCH (2, 3, 13–15). In this case, granulomatous lesions in the dog’s lungs were negative for CD90, FXIIIa, and e-cadherin, findings consistent with NLCH. Notably, alveolar histiocytes showed marked CD90 and FXIIIa positivity, likely reflecting an inflammatory state or endothelial cell activation at the alveolar level. The negative labeling of CD90 and FXIIIa in granulomatous lesions surrounding airways likely reflects differences in the immunophenotype and functional specialization of macrophages involved in granuloma formation versus those in the alveolar regions. Granulomatous lesions are dominated by epithelioid macrophages and multinucleated giant cells, which may downregulate CD90 and FXIIIa during differentiation. This suggests that the granulomatous inflammation in the airways involves distinct cellular pathways and stimuli compared to the alveolar inflammation. Furthermore, the absence of S-100 protein expression significantly reduces the likelihood of LCH, as S-100 positivity is a common feature of LCH (6, 11, 16). These findings suggest that the lung lesions represent a dysregulated inflammatory response to an unknown stimulus, supporting the diagnosis of NLCH.

Furthermore, there was also marked expression of the infiltrating cells with CD204 and IBA-1, both of which are supportive of the infiltrating cells’ macrophage origin. Briefly, CD204, also known as scavenger receptor A (SRA), is a cell surface receptor primarily expressed on macrophages. It plays a role in phagocytosis and clearance of modified or foreign substances. Whereas IBA-1 (ionized calcium-binding adapter molecule 1), also known as AIF-1 (allograft inflammatory factor 1), is another marker used to identify macrophages and microglial cells. Positive labeling for IBA-1 indicates the presence of these cell types, suggesting an activated state or an inflammatory response. In veterinary medicine, CD204 monoclonal antibody has been reported to recognize canine tissue-resident macrophages, including alveolar macrophages (17). However, in a few studies, the combination of both IBA-1 and CD204 positive in canine lung samples were found to be strongly associated with histiocytic sarcomas, a malignant neoplasm of histiocytic origin, in various organs of dogs (18, 19). In this case, the diagnosis of histiocytic sarcoma was excluded based on the absence of histologic evidence of malignancy. The infiltrates exhibited a bland cytologic appearance, with no mitotic figures, anisocytosis, or anisokaryosis appreciated. Additionally, the distribution of the infiltrates was limited to the pulmonary parenchyma and lacked widespread or multi-organ involvement, which is typically observed in systemic histiocytosis or histiocytic sarcoma. Moreover, CD204 expression has been reported as variable in feline histiocytic diseases, canine reactive histiocytoses, and canine systemic histiocytoses, rendering definitive differentiation between neoplastic and non-neoplastic lesions not possible solely on this marker (20). Canine reactive cutaneous histiocytosis is typically confined to the skin and draining lymph nodes, while systemic histiocytosis involves skin, extracutaneous sites, or both (1, 2). In both cases, dermal lesions are a principal clinical feature (1, 2). Comparatively, this case had no dermal manifestations of granulomatous lesions, further supporting that this case does not align with reactive or systemic histiocytosis. The findings collectively suggest that this case represents a reactive, localized histiocytic process rather than a systemic or malignant histiocytic condition.

We considered other pulmonary interstitial diseases such as eosinophilic pneumonia, pulmonary alveolar proteinosis, diffuse alveolar hemorrhage, lipid/lipoid pneumonia, pulmonary hyalinosis, pulmonary alveolar microlithiasis, and histiocytic sarcoma complex as potential differential diagnoses. However, none of these conditions were histologically confirmed in this case. In fact, histology was more suggestive of an idiosyncratic inflammatory or immune-mediated condition. To elaborate, the combination of the (1) severe cellular infiltrate centered on medium to small bronchioles, (2) presence of a bronchiolocentric infiltrate comprised mostly of CD204, IBA-1, CD163, CD90, FXIIIA-positive large reactive alveolar macrophages, (3) replacement of the mucosa by macrophages, (4) occasional presence of similar infiltrates as nodules not centered on bronchioles, (5) significant lymphoplasmacytic infiltrate in the surrounding parenchyma, including around blood vessels and affected bronchioles, infiltration of lymphocytes and plasma cells into the pleura; and (6) mild to moderate fibrosis in this case are more consistent with the reported NLCH in people, ECD (12, 21, 22). Thus, the negative expression of e-cadherin, CD90, FXIIIa, CD163 in the granulomatous lung lesions, the alveolar macrophagic immunoreactivity of CD90, CD163, FXIIIa, CD204, and IBA-1 are most consistent with an acute, dysregulated inflammatory insult to the pulmonary parenchyma.

Additionally, pyogranulomatous bronchopneumonia was considered; however, the histologic findings in this case—particularly the airway-oriented, macrophage-dominant infiltrates, the marked absence of infectious organisms, and the lack of widespread degenerative neutrophilic inflammation—strongly argue against this diagnosis. Pyogranulomatous processes typically feature a predominant neutrophilic response, with infectious agents such as bacteria or fungi often serving as inciting causes. In this case, despite extensive diagnostic testing—including special stains and PCR for bacterial, fungal, and viral pathogens—no infectious organisms were identified. Furthermore, the airway-oriented macrophage population, characterized by CD204 and IBA-1 positivity, represents a distinct histopathologic feature that is not typically observed in bronchopneumonia, where neutrophilic inflammation predominates. Additionally, the absence of e-cadherin and CD90 expression in granulomatous lesions helps differentiate NLCH from other histiocytic conditions, including LCH and other reactive or systemic histiocytoses. This immunophenotypic profile is consistent with findings in human NLCH, which showcases the importance of IHC in defining and differentiating histiocytic diseases.

Finally, this dog could also be considered an AIP, a type of pneumonia that has not yet been reported in veterinary medicine (21). In people, AIP is characterized by respiratory disease of less than 60 days’ duration, bilateral and diffuse infiltrates on imaging studies, histopathologic features of severe alveolar damage (including temporal homogeneity of lesions), a lack of a known inciting event or predisposing condition, and no prior abnormal thoracic radiographs (23). With regard to this dog’s case, the timeline of clinical progression of less than 21 days, diagnostic imaging consistent with bilateral and diffuse infiltrates, lack of a known inciting event or pre-existing pulmonary disease, normal thoracic radiographs 2 months prior to the event, and the histologic evidence of severe alveolar and interstitial damage—though not classic diffuse alveolar damage (DAD)—fulfill the criteria for a concurrent diagnosis of AIP. This case demonstrates a rapid, idiopathic inflammatory response, aligning with the acute and fulminant nature of AIP, further supporting the hypothesis that PNLCH could be considered a subset or manifestation of idiopathic interstitial pneumonia in veterinary species.

Regardless of its classification, the prognosis and effectiveness of treatment options for PNLCH in veterinary medicine remain unclear due to the scarcity of published data and the absence of established protocols. However, in cases of non-malignant histiocytoses, current management focuses on supportive care, including mechanical ventilation, oxygen therapy, and empirical immunosuppressive therapies such as corticosteroids though, these cases eventually succumb due to the disease progression (1, 2). Although therapies such as methotrexate, canakinumab, vemurafenib, and interleukin-1 inhibitors have been explored in people with NLCH or Erdheim-Chester disease (ECD) (8, 24), these treatments are not directly translatable to veterinary cases due to significant differences in molecular pathogenesis, such as the absence of activating MAPK mutations in canine cases. Additionally, the challenges of antemortem diagnosis further complicate treatment. Advanced diagnostic tools like genomic sequencing, while common in people (6, 7), are underutilized in veterinary medicine due to high costs and limited accessibility. Developing cost-effective genomic tools and expanding histopathologic and immunohistochemical profiling for canine PNLCH are critical to improving diagnostic accuracy. Furthermore, additional cases with similar IHC profiles are needed to establish treatment protocols and refine prognostic indicators. Moreover, in cases of ARDS-like syndromes or idiopathic interstitial pneumonia in veterinary patients, supportive care remains the cornerstone of treatment. The parallels drawn between feline PLCH and this case highlight the potential for antemortem diagnosis through bronchoalveolar lavage, cytology, and immunocytochemical staining (25). Thus, additional cases which showcase a similar IHC profile to this dog’s case are needed which will likely require biopsies and histopathologic review. This approach, combined with ongoing research into empirical treatment options, holds promise for improving the quality of life and survival outcomes for affected patients.

## Conclusion

NLCH is a rare condition with varied clinical presentations, posing significant diagnostic challenges. This report describes a case of PNLCH in a dog with progressive respiratory failure, whose clinical progression closely resembles other NLCH cases in people. The case aligns most closely with AIP based on clinical course, imaging, and histologic findings, while also exhibiting granulomatous inflammation and an immunophenotypic profile akin to ECD. This suggests a novel presentation of PNLCH with overlapping features of AIP, distinguishing it from both ECD and classic AIP. Although definitive ante-mortem diagnosis is difficult in veterinary medicine, key indicators—such as insidious onset of respiratory signs, rapid progression, peripheral pulmonary nodules on imaging, non-responsiveness to antimicrobial and bronchodilator therapies, and characteristic cytologic and histologic findings—may support a pre-mortem consideration of PNLCH. Further studies are essential to refine its classification, explore its relationship to other interstitial and histiocytic diseases, and improve diagnostic and management strategies.

## Data Availability

The original contributions presented in the study are included in the article/Supplementary material, further inquiries can be directed to the corresponding author.

## Supplementary material

SUPPLEMENTARY MATERIAL FIGURE 1 Dorsal CT view of the thorax highlighting mild tracheobronchial lymph node enlargement (arrows). The arrows point to the enlarged tracheobronchial lymph nodes positioned near the trachea and mainstem bronchi. This lymphadenopathy may be indicative of underlying pathology, such as inflammation or neoplastic disease, contributing to the respiratory abnormalities observed in this patient.

## References

[1] Moore PF. Histiocytic diseases. Vet Clin. (2023) 53:121–40. doi: 10.1016/j.cvsm.2022.07.010, PMID: 36270835

[2] Moore PF. Histiocytic proliferative diseases of dogs and cats. Schalm’s veterinary. Hematology 7th edition. (2022):633–48. doi: 10.1002/9781119500537.ch74, PMID: 39908183

[3] Argenta FF de Britto FC Pereira PR Rissi DR Gomes C da Costa FVA. Pulmonary Langerhans cell histiocytosis in cats and a literature review of feline histiocytic diseases. J Feline Med Surg. (2020) 22:305–12. doi: 10.1177/1098612X19842384, PMID: 30977699 PMC10814658

[4] Fulmer AK Mauldin GE. Canine histiocytic neoplasia: an overview. Can Vet J. (2007) 48:1041. PMID: 17987966 PMC1978291

[5] Leissinger M Garber J Fowlkes N Grooters A Royal A Gaunt S. *Mycobacterium fortuitum* lipoid pneumonia in a dog. Vet Pathol. (2015) 52:356–9. doi: 10.1177/0300985814531497, PMID: 24788402

[6] Emile J-F Abla O Fraitag S Horne A Haroche J Donadieu J. Revised classification of histiocytoses and neoplasms of the macrophage-dendritic cell lineages. Blood J Am Soc Hematol. (2016) 127:2672–81. doi: 10.1182/blood-2016-01-690636 PMC5161007 26966089

[7] Classen C Minkov M Lehrnbecher T. The non-Langerhans cell histiocytoses (rare histiocytoses)–clinical aspects and therapeutic approaches. Klin Padiatr. (2016) 228:294–306. doi: 10.1055/s-0042-109713, PMID: 27846659

[8] Barros MS Krupa BS Madineni MD Kenyon MD Lawrence C Farrell MD. A rare case of a systemic non-Langerhans Histiocytosis presenting with diabetes insipidus and a tentorial mass. JHN J. (2015) 10:3. doi: 10.29046/JHNJ.010.1.004

[9] Wang JN Wang FD Sun J Liang ZY Li J Zhou DB. Pulmonary manifestations of Erdheim–Chester disease: clinical characteristics, outcomes and comparison with Langerhans cell histiocytosis. Br J Haematol. (2021) 194:1024–33. doi: 10.1111/bjh.17712, PMID: 34423426

[10] Braiteh F Boxrud C Esmaeli B Kurzrock R. Successful treatment of Erdheim-Chester disease, a non–Langerhans-cell histiocytosis, with interferon-α. Blood. (2005) 106:2992–4. doi: 10.1182/blood-2005-06-2238, PMID: 16020507

[11] Weitzman S Jaffe R. Uncommon histiocytic disorders: the non-Langerhans cell histiocytoses. Pediatr Blood Cancer. (2005) 45:256–64. doi: 10.1002/pbc.20246, PMID: 15547923

[12] Rush WL Andriko JAW Galateau-Salle F Brambilla E Brambilla C Ziany-bey I. Pulmonary pathology of Erdheim-Chester disease. Mod Pathol. (2000) 13:747–54. doi: 10.1038/modpathol.3880130, PMID: 10912934

[13] Rissi DR Brown CA Gendron K Good J Lane S Schmiedt CW. Pancreatic Langerhans cell histiocytosis in a cat. J Vet Diagn Invest. (2019) 31:859–63. doi: 10.1177/1040638719874857, PMID: 31510879 PMC6900718

[14] Busch M Reilly C Luff J Moore PF. Feline pulmonary Langerhans cell histiocytosis with multiorgan involvement. Vet Pathol. (2008) 45:816–24. doi: 10.1354/vp.45-6-816, PMID: 18984784

[15] Hernández-San Martín M Vargas-Mora P Aranibar L. Juvenile xanthogranuloma: an entity with a wide clinical spectrum. Actas Dermosifiliogr (Engl Ed). (2020) 111:725–33. doi: 10.1016/j.ad.2020.07.004, PMID: 32721389

[16] Kobayashi M Tojo A. Langerhans cell histiocytosis in adults: advances in pathophysiology and treatment. Cancer Sci. (2018) 109:3707–13. doi: 10.1111/cas.13817, PMID: 30281871 PMC6272080

[17] Tomokiyo R-i Jinnouchi K Honda M Wada Y Hanada N Hiraoka T. Production, characterization, and interspecies reactivities of monoclonal antibodies against human class a macrophage scavenger receptors. Atherosclerosis. (2002) 161:123–32. doi: 10.1016/S0021-9150(01)00624-4, PMID: 11882324

[18] Kato Y Murakami M Hoshino Y Mori T Maruo K Hirata A. The class a macrophage scavenger receptor CD204 is a useful immunohistochemical marker of canine histiocytic sarcoma. J Comp Pathol. (2013) 148:188–96. doi: 10.1016/j.jcpa.2012.06.009, PMID: 22901707

[19] Thongtharb A Uchida K Chambers JK Kagawa Y Nakayama H. Histological and immunohistochemical studies on primary intracranial canine histiocytic sarcomas. J Vet Med Sci. (2016) 78:593–9. doi: 10.1292/jvms.15-0627, PMID: 26668164 PMC4873849

[20] Hirabayashi M Chambers JK Sumi A Harada K Haritani M Omachi T. Immunophenotyping of nonneoplastic and neoplastic histiocytes in cats and characterization of a novel cell line derived from feline progressive histiocytosis. Vet Pathol. (2020) 57:758–73. doi: 10.1177/0300985820953538, PMID: 32885737

[21] Reinero C. Interstitial lung diseases in dogs and cats part I: the idiopathic interstitial pneumonias. Vet J. (2019) 243:48–54. doi: 10.1016/j.tvjl.2018.11.010, PMID: 30606439

[22] Reinero C. Interstitial lung diseases in dogs and cats part II: known cause and other discrete forms. Vet J. (2019) 243:55–64. doi: 10.1016/j.tvjl.2018.11.011, PMID: 30606440

[23] Vourlekis JS Brown KK Schwarz MI. Acute interstitial pneumonitis: current understanding regarding diagnosis, pathogenesis, and natural history. InSeminars in respiratory and critical care medicine 2001. New York, NY, USA: Thieme Medical Publishers, Inc., 333 Seventh Avenue. (2001). 22:399–408. 10.1055/s-2001-17383 16088688

[24] Hundal J Bowers D Gadela NV Jaiswal A. Rare case of refractory hypoxia and severe multiorgan failure from secondary Lymphohistiocytosis successfully bridged to treatment with extracorporeal membrane oxygenation support. Indian J Crit Care Med. (2022) 26:970–3. doi: 10.5005/jp-journals-10071-24284, PMID: 36042774 PMC9363810

[25] Mau A Chiu ES Armien A Johnson LR Moore PF Vernau W. Antemortem cytologic diagnosis of pulmonary Langerhans cell histiocytosis in a cat. Vet Clin Pathol. (2023) 52:691–7. doi: 10.1111/vcp.13304, PMID: 37914537

