# Supplementary figures and images for: Case report: Pulmonary non-Langerhans cell histiocytosis in a dog with acute interstitial granulomatous pneumonia
Source: Front Vet Sci. 2025 Feb 25;12:1522119. doi: 10.3389/fvets.2025.1522119 (PMC11893815; doi:10.3389/fvets.2025.1522119)

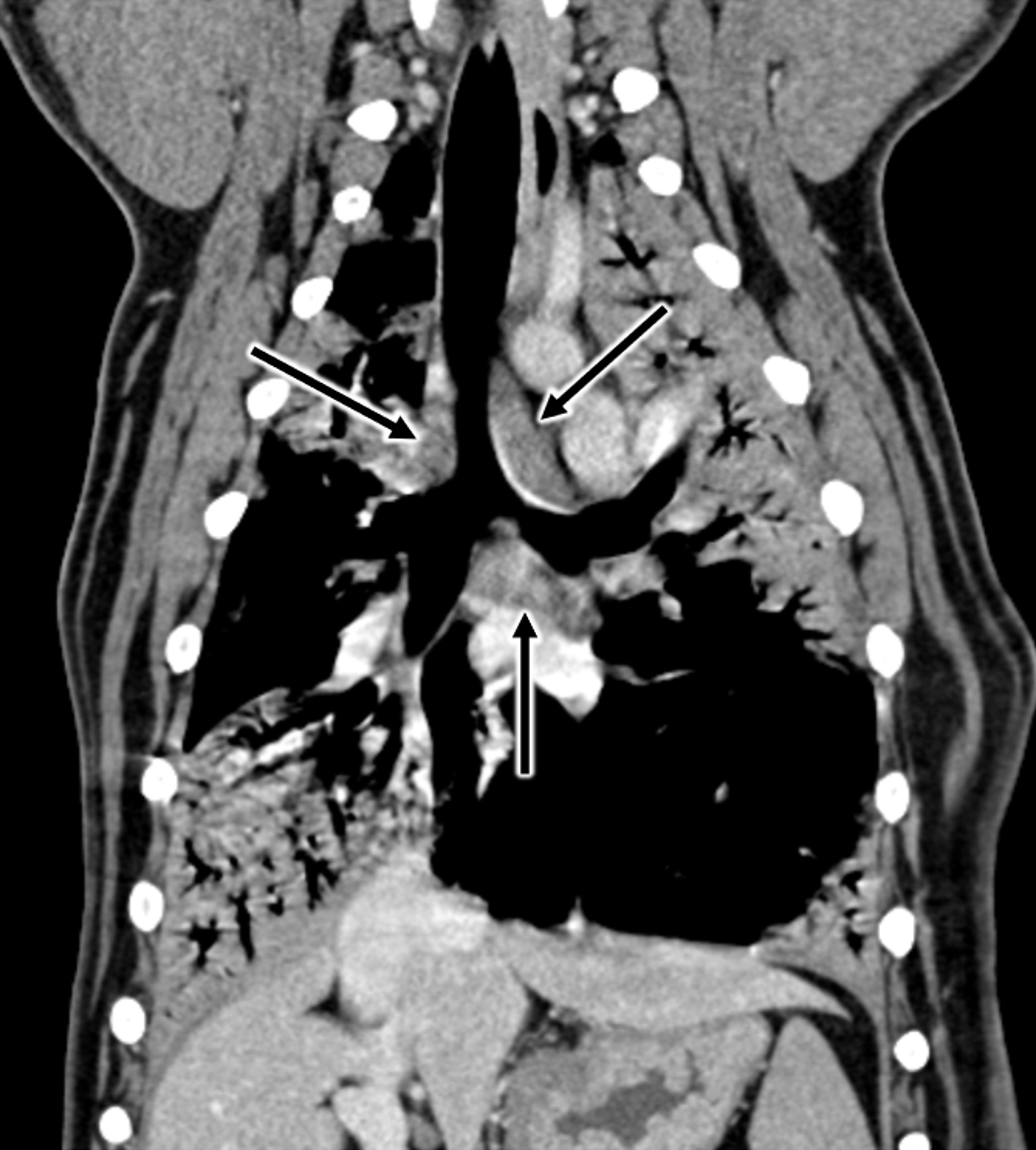

Supplement: SUPPLEMENTARY MATERIAL FIGURE 1 — Dorsal CT view of the thorax highlighting mild tracheobronchial lymph node enlargement (arrows). The arrows point to the enlarged tracheobronchial lymph nodes positioned near the trachea and mainstem bronchi. This lymphadenopathy may be indicative of underlying pathology, such as inflammation or neoplastic disease, contributing to the respiratory abnormalities observed in this patient. [file Image_1.tiff]
