# Supplementary material for: Case report: Pulmonary non-Langerhans cell histiocytosis in a dog with acute interstitial granulomatous pneumonia
Source: Front Vet Sci. 2025 Feb 25;12:1522119. doi: 10.3389/fvets.2025.1522119 (PMC11893815; doi:10.3389/fvets.2025.1522119)
Supplement: Supplementary file 2 [file Table_1.docx]

| **Supplemental Table 1. List of special and immunohistochemical stains performed on the lungs of an 8-year-old male, intact Rottweiler with pulmonary non-Langerhans cell histiocytosis.** | | | | | |
| --- | --- | --- | --- | --- | --- |
| **SPECIAL / IHC STAINS** | **HISTOLOGIC UTILITY** | **LOCATION OF REACTIVITY** | | | **FINDINGS / INTERPRETATIONS** |
|  |  | **ALVEOLI** | **PULMONARY PARENCHYMA** | **CELLULAR INFILTRATE** |  |
| **CD163** | **Highlights monocytes / macrophages, histiocytic tumors** | **+** | **–** | **–** | **Positive identification / labeling of tissue resident macrophages** |
| **CD204** | **Highlights tissue resident macrophages** | **+** | **–** | **–** | **Positive identification of epithelial and alveolar (tissue resident) macrophages** |
| CD3 | Highlights T-cells | – | – | – | Ruled out neoplastic and non-neoplastic, T-cell associated disorders |
| **CD90** | **Highlights immature hematopoietic stem cells, neurons, connective tissue, activated endothelial cells, fibroblasts (variable)** | **+** | **–** | **–** | **Positive identification / labeling of alveolar histiocytes and those which did not stain were consistent with non-alveolar macrophages** |
| CDV IHC | Highlights inclusion bodies associated with Canine Distemper Virus | – | – | – | Ruled out canine distemper virus |
| E-cadherin | Highlights Langerhan cells | – | – | – | Lack of immunolabeling used to differentiate Langerhans cell etiology from the robust histiocytosis |
| **Factor XIIIa** | **Highlights fibrohistiocytic proliferations, macrophages associated with an adaptive inflammatory reaction** | **+** | **+** | **–** | **Positive identification / labeling of alveolar macrophages** |
| Giemsa | Detects mast cells and microorganisms, such as Giardia or Helicobacter | – | – | – | Ruled out infectious etiologic agents |
| GMS | Detects fungi, some bacteria, mycobacteria, *Strongyloides spp*., and some viral inclusion bodies | – | – | – | Ruled out infectious etiologic agents |
| **IBA-1** | **Highlights macrophages associated with immune-mediated or inflammatory diseases** | **+** | **+** | **–** | **Positive identification of epithelial and alveolar (tissue resident) macrophages** |
| Luna | Highlights melanocytes and microsporidian spores | – | – | – | Ruled out infectious etiologic agents |
| PAS | Detects some bacterial microorganisms, amorphous or granular globules in lungs, and some fungal cell walls | – | – | – | Ruled out infectious etiologic agents |
| PAX-5 | Highlights B-cells | – | – | – | Ruled out neoplastic and non-neoplastic, B-cell associated disorders |
| S-100 protein | Highlights cells associated with melanoma, nerve sheath tumors, clear cell sarcoma of soft tissue, LCH, RDD, glial tumors and myoepithelial tumors | – | – | – | Lack of immunolabeling was used to further rule-out Langerhans cell histiocytosis, human Rosai-Dorfman's disease, and other neoplastic histiocytic disorders |
| Silver | Highlights hyphae and spores of fungi | – | – | – | Ruled out infectious etiologic agents |
| Site-Acid Fast | Detects acid-fast staining microorganisms, including those that are partially acid-fast staining | – | – | – | Ruled out infectious etiologic agents |
| **VVG** | **Highlights elastic lamina** | **+** | **+** | **–** | **Highlighted the pulmonary elastic lamina and confirmed infiltrate predilection towards bronchioles** |
| Warthin-Starry | Detects *Helicobacter pylori*, *Bartonella* spp, and other spirochetes | – | – | – | Ruled out infectious etiologic agents |
| ZNAF | Detects acid-fast staining microorganisms | – | – | – | Ruled out infectious etiologic agents |
| *+ = positive; – = negative; CDV = Canine Distemper Virus; GMS = Grocott-Gömöri methenamine Silver; PAS = Periodic Acid-Schiff CD = Cluster of differentiation; IBA-1 = Ionized calcium-binding adapter molecule 1; H&E = Hematoxylin and Eosin; VVG = Verhoeff van Gieson; PAX5 = Paired Box 5; LCH = Langerhans's Cell Histiocytes; iDC = Interstitial Dendritic Cells; RDD = Rosai-Dorfman Disease; ZNAF = Ziehl Neelsen Acid Fast* | | | | | |
