# Supplementary material for: Case report: Pulmonary non-Langerhans cell histiocytosis in a dog with acute interstitial granulomatous pneumonia
Source: Front Vet Sci. 2025 Feb 25;12:1522119. doi: 10.3389/fvets.2025.1522119 (PMC11893815; doi:10.3389/fvets.2025.1522119)
Supplement: Supplementary file 3 [file Table_2.docx]

| **Supplemental Table 2.** Comparison of pulmonary histology findings in a canine case and human non-Langerhans cell histiocytosis, including histological, immunohistochemical, and clinical features. | | |
| --- | --- | --- |
| **Feature** | **Current Case Canine Pulmonary Histology** | **Human Non-Langerhans Cell Histiocytosis (NLCH)** |
| Primary Inflammatory Cells | Macrophage-rich infiltrates intermixed with lymphocytes, primarily centered on bronchioles | Macrophage (histiocyte)-rich infiltrates, often foamy, primarily distributed in pleura, septa, and perivascular interstitium |
| Distribution of Infiltrate | Found mainly in bronchioles, alveolar interstitium, pleura, and surrounding vasculature | Distributed primarily in pleura, septa, and perivascular interstitium, often in a lymphangitic pattern |
| Pattern of Infiltrate | Multifocal with mild to moderate fibrosis | Lymphangitic with associated fibrosis |
| Inflammatory Components | Lymphoplasmacytic inflammatory infiltrates | Lymphoplasmacytic inflammatory infiltrates |
| Fibrosis | Mild to moderate fibrosis distributed multifocally; no evidence of end-stage fibrosis | Fibrosis associated with histiocytic infiltrates; variable extent depending on subtype |
| Temporal Homogeneity | Lesions show temporal homogeneity; no evidence of end-stage fibrosis | Temporal homogeneity varies; active and fibrotic lesions may coexist |
| IHC Staining Characteristics | CD204 + IBA-1 +  CD1a – E-cadherin –  CD90 +  PAX-5 –  CD3 – | CD68 +  CD1a –  Additional markers vary by subtype |
| Clinical Presentation | Respiratory distress with coughing; progressive signs in advanced cases | Dyspnea, cough, and systemic symptoms (e.g., fever, weight loss); systemic involvement may occur |
| Radiographic Findings | Diffuse interstitial to nodular patterns; multifocal opacities; some regions mimic mass-like lesions | Interstitial thickening or nodules; imaging findings can vary based on subtype |
| Prognosis | Poor with extensive pulmonary involvement; variable response to therapy | Prognosis depends on subtype; aggressive forms (e.g., Erdheim-Chester disease) are associated with poor outcomes |
| *+ = Positive; – = Negative; Acid-Fast = Ziehl-Neelsen Acid Fast; CD = Cluster of Differentiation; CDV = Canine Distemper Virus; E-Cadherin = Epithelial Cadherin; Giemsa = Giemsa stain for bacteria; GMS = Grocott-Gömöri Methenamine Silver; Gram = Gram stain for bacteria classification; H&E = Hematoxylin and Eosin; IBA-1 = Ionized Calcium-Binding Adapter Molecule 1; iDC = Immature Dendritic Cells; LCH = Langerhans Cell Histiocytosis; Luna = Luna stain for melanocytes and elastin; PAX-5 = Paired Box 5; PAS = Periodic Acid-Schiff; Temporal Homogeneity = Lesions are at the same stage of development; Thy-1 = CD90, a marker for immature dendritic cells; VVG = Verhoeff-Van Gieson stain for elastic lamina.* | | |
